# A Commentary on “Lactated Ringer’s solution versus saline fluid resuscitation for reducing progression to moderate-to-severe acute pancreatitis: a systematic review and meta-analysis”

**DOI:** 10.1097/JS9.0000000000002923

**Published:** 2025-07-04

**Authors:** Xiangqing Liu, Hongying Xing, Rong Zhou

**Affiliations:** a Department of Burn and Orthopedics, Joint Logistics Force 969 hospital, Hohhot, Inner Mongolia Autonomous Region, China; b Department of Nursing, Joint Logistics Support Force 969 hospital, Hohhot, Inner Mongolia, China; c Supply Room, 969th Hospital of Joint Logistics Support Force, Hohhot, Inner Mongolia Autonomous Region, China

*Dear Editor,*

We read with great interest the recent article titled “Lactated Ringer’s solution versus saline fluid resuscitation for reducing progression to moderate-to-severe acute pancreatitis: a systematic review and meta-analysis”^[1]^ published in the International Journal of Surgery. The purpose of this study is to clarify the differences between normal saline (NS) and lactated Ringer’s solution (LR) in the initial management of AP. The outcome demonstrated that LR can considerably delay the onset of moderate to severe pancreatitis. Furthermore, LR is linked to a shorter total hospital stay, a lower incidence of local complications, and a decreased requirement for intensive care unit hospitalizations, all of which contribute to a better clinical outcome. Other difficulties, however, showed no discernible changes. Although more research is required to confirm these outcomes, subgroup analyses also point to LR’s ability to reduce pancreatic necrosis and other indices. Future studies must, however, address a number of important concerns.

First, the study design’s limitations. The study primarily relies on a mix of data from randomized controlled trials (RCTs) and observational studies. While this increases the sample size, it also introduces potential heterogeneity and bias. Although the study adheres to the PRISMA and AMSTAR guidelines, the design of the observational studies introduces confounding factors, such as baseline patient characteristics and differences in treatment regimens, which may affect the reliability of the results. Moreover, the study does not clearly specify how these confounding factors are controlled, particularly when adjusting for variables. Some studies only provide unadjusted data, further diminishing the credibility of the conclusions. Additionally, the study fails to stratify the included RCTs and observational studies, which could lead to inconsistent results.

Second, consider the problem of sample heterogeneity. The study included 10 trials, covering various regions, patient groups, and treatment methods, leading to a high degree of sample heterogeneity. For instance, some studies focused on mild acute pancreatitis (MAP), while others included moderate to severe cases (SAP). Despite subgroup analyses, heterogeneity remained, such as the *I*^2^ = 52% for hospital stay analysis, suggesting that the results might be influenced by unmeasured variables. Additionally, some studies had small sample sizes (e.g., *n* = 19), which may not adequately represent the overall population.

Third, the secondary outcome’s statistical power was inadequate. The analysis of secondary outcomes, such as mortality, organ failure, and SIRS, showed no significant differences. However, the sample sizes for these results were small, leading to insufficient statistical power. For instance, the mortality analysis included only seven studies with a limited number of events (9 in the LR group vs. 27 in the NS group), making it difficult to draw reliable conclusions. Additionally, the analysis of SIRS at 24, 48, and 72 hours did not reveal significant differences, which may be due to variations in measurement methods and patient baseline characteristics.

In summary, this study systematically analyzed the effects of LR and NS in the fluid resuscitation of acute pancreatitis, but it had design limitations, sample heterogeneity, and inconsistent fluid regimens. Future studies should adopt larger-scale, standardized RCTs, incorporating inflammatory markers, etiological stratification, and long-term outcomes to further validate the superiority of LR^[2,3]^. All this was observed regarding the transparency in the reporting of Artificial Intelligence—the TITAN guideline^[4]^.

## Data Availability

No data was used in this Letter to the Editor.
